# Quarterly Summary

**Published:** 1854-07

**Authors:** 


### QUARTERLY SUMMARY.

### DENTAL SCIENCE.

1.—*A new Non-conductor for Capping Dental Nerves or Tender Teeth.*—In the Dental News Letter for April, 1854, Dr. C. A. Du Bouchet says: "With a view to obviate the evils arising from filling a tooth over an exposed nerve, or in a tender state, various operations have been devised and performed. The operation of capping the nerve of a tooth was, I believe, prior to that of destroying it; of course I now allude to the ancient procedure of the actual cautery. Capping nerves has never, as far as I can ascertain, proved an eminently successful operation. This want of success, attributable to a variety of causes, (to which I shall presently allude,) gave rise to the employment of caustics and escharotics to cause the death of that troublesome subject."

Arsenious acid then came into use—but as this was yet involved in darkness, the use of that agent and the subsequent treatment, did not meet the requirements of the case, and thus the failures being numerous, compelled the practitioner to abandon it for the time. The doctor then remarks, that the profound researches of contemporaries, and successful experiments, have entirely settled this point, and that it is generally believed we know how to use arsenious acid; that we know the dead pulp should be removed, and a time elapse ere we fill a tooth; but in this we have lost sight of the opposite operation, of preserving an exposed nerve in a *healthy condition.*

The doctor states, that on this point he occupies no half condition—that he has no faith in the operation which will merely allow a tooth to stay in the mouth. He then proposes to present to the profession one *new substance,* which, to his knowledge, has never been used for this purpose. He believes that the cases are unfrequent where capping would be advisable; that if a nerve became exposed by the decay of a tooth, or has seriously been wounded in excavating a carious tooth, it were worse than useless to attempt capping in any uncertain cases, and that he prefers to follow Prof.

White's indications for the use of arsenic. Dr. Du B. here gives cases where capping is advisable; he also shows to what causes the want of success is attributable, and he is satisfied, that although the difficulties are great, if the operation were performed in any of the cases he points out as practiced by him, that it will be successful in ninety-nine cases out of a hundred. Dr. Du B., after showing the inexpediency of any of the materials used for capping nerves, viz. gold, lead, the different gums, asbestos, pulverized glass, feldspar, plaster of paris, &c., but found that none of them were so well calculated to fulfil the requirements as *horn.* It can be pared to any size—can be obtained of any hue; it is a good non-conductor, and is indestructible. He says the horn can be used to great advantage in filling front teeth where they are decayed to a shell, by placing a thin plate against the front enamel, the natural color is preserved. In remarking upon the paper of Dr. Du Bouchet, the editor of the News Letter says:

"The above intelligent paper is of much interest to the profession, from the fact that the author seems to have given it great attention for many years; and especially to those who believe it is sound or good practice to endeavor to save an exposed pulp alive. Medicating an exposed pulp, and capping or arching it over, in order to prevent the actual contact of external agents, seems to have been, until of late years, the principal means depended upon to obtain so desirable an end; but this method of treatment has given way to apparently a very different theory and practice. *Non-conductors* and non-metallic substances, seem now to be relied on entirely. Many in the profession are doubtless familiar with most of the substances named, and each one, in their turn, have had their advocates. But horn and shell seem, and very justly, to be preferred by the author, and which is favorably brought before the profession. It will take the shape of the bottom of the cavity much better, we have no doubt, than many other articles used heretofore, especially if it be prepared as directed, and is as good, if not a better non-conductor, than any other substance named, that is as close in texture. The profession had been apprised for some time, by one of two eminent practitioners, that they were experimenting with a substance which, if placed between the plugging material and the exposed pulp, there would be no danger of unpleasant consequences, and which experiments, when perfected or completed, or satisfactory to them, would be communicated to the profession. We waited patiently for the revelation of *Goose-Quill,* and by some apparently informal manner, it became known to many of the profession, but not as yet in that desirable form that could be wished, for the credit of the original claimant of the suggestion of the substance. It does not seem to us that the question turns upon the difference between the capacities of these different substances to resist the changes of temperature; but upon the pathological condition of the pulp, when actually exposed, it does not seem possible that any foreign substance can take the place in any degree of a normal tissue, especially to be in close contact with so impressible a tissue as a dental pulp. We have said elsewhere, and we have as yet observed nothing to cause us to change our position, that as well might we expect to procure a healthy function of the *rete mucosum,* when denuded of the *epidermis,* by substituting one of our own invention, as to procure a healthy function of the pulp when deprived of its natural protection—the bone. The various modes of treatment which have for their object the preservation of the pulp, must be of that order. We regard the propriety of capping an entirely exposed nerve as already settled, and the intervention of any substance to cut off the effects of external influence of any agents differing from the material used as a plug, as of questionable propriety. The uncomfortable impression of cold upon a tooth where there is a due amount of dentine to keep up a healthy function of the pulp, is always of short duration, and when that has passed off, the tooth is in a better condition, plugged with one material, than with more and of different characters. And where the remaining plate of dentine is extremely thin, it is as liable to be absorbed or to go on to soften, as to become hardened or increase in thickness."

2.—*A Freak of Nature.*—A writer in the News Letter for April, 1854, signing himself H., mentions a case in which, upon opening an enlargement of the palatine surface in the mouth of a lady aged fifty, discovered a beautiful canine tooth. "The peculiar position," he says, "was an inch and a half from the alveolar border, five or six lines to the right of the mesial line, and presenting from within outwards, so that a pair of straight, narrow beaked forceps, was employed for its removal."

3.—*Crystallized and Sponge Gold.*—Dr. C. W. Ballard, in addressing the editors of the News Letter, in the last April number, upon the subject of *crystallized and sponge gold*, says, "I wish, through the columns of your journal, to call the attention of the profession to the preparations of crystallized and sponge gold lately introduced for the purpose of filling teeth. I have been for some months testing and experimenting with them, and from the results, think myself warranted in recommending the new preparations, as being of the utmost importance to the operating dentist.

The same force will make a harder and more solid filling of it than if gold foil were used. Its plasticity enables the operator to fill shallow or saucer-shaped cavities with greater ease, thus doing away, in a great measure, with the necessity of removing sound dentine or enamel, in order to find a sufficient hold or stay for the ordinary filling. This I consider of great importance, as it leaves so much more of the natural organs; in fact, there is just as much more of the tooth saved. If it is the professed object of the dentist to save teeth, he should save all the teeth he can, and also as much of any one tooth as the best means in his power will enable him to preserve for the benefit of his patient. All the dentine or enamel thus saved can be considered as an advance in our practice, and be it little or much, the mere ability to save it by means of this preparation, constitutes, in my opinion, a most powerful claim in its favor.

The ease with which separate masses of gold are welded together under the condensing instruments, is another valuable property. It enables the operator, after thoroughly packing a thin layer in the bottom of the cavity, to pack another layer upon it. The two, uniting completely, forming, if properly worked, a solid mass of the metal.

These layers can thus be added until the whole cavity is filled, by what is thus rendered one solid piece of gold, as solid and compact at the bottom, sides and centre, as it is upon the surface. Of course, a filling possessing these requisites should finish up well, and this can be really finished beautifully, though in this respect it will not surpass foil when worked by skillful hands, unless it may be in the durability of its brilliance, which I believe will prove to be the case in consequence of the greater solidity of the mass.

These preparations are admirably calculated for filling compound cavities. Where decay has extended to more than one side or surface of the tooth, such cavities can be filled and the form of the tooth restored with greater ease and security than with foil. The gain here is in the time, labor, and in the durability of the operation, as also in the occasional saving of tooth substance, in consequence of the diminished necessity of using the file.

"Another advantage yet unmentioned is no slight one. It is always ready to be put into the cavity; it has only to be broken into small pieces of suitable size. Hence, the time spent in cutting, rolling, folding, or twisting the foil, can be more profitably employed.

"To sum up; its claims for professional favor, are:—1st, a saving of labor, of which most men are not too fond. If any are, they have 2d, a saving of time, and can consequently accomplish more in a day, if they wish. 3d, a saving of tooth substance, in fact, of teeth. It is for this object we practice. 4th and last, and greatest, a better operation when completed.

"I now propose to notice some objections I have heard raised against its adoption. They are more fallible than numerous—some are very trifling, though seriously urged, viz.—1st, *it is too expensive;* 2d, there is too great waste; 3d, it will look badly in a front tooth when the walls of the cavity are composed only of enamel; 4th, a dentist will have to learn to fill teeth over again; 5th, an entire change of instruments will be required.

"When I state that these are all the objections that have come to my ear, and I have heard the opinions of quite a number of dentists expressed, I believe many who may read this article, and who have hitherto considered the new preparation a humbug, will at once give it some attention. I most earnestly beg of them to do so, and hope they will give the profession the benefit of their experience. As the above objections have been seriously urged, I will endeavor to give them serious and satisfactory refutation.

"As to the first, the cost, per ounce, is about the same as the best quality of gold foil. The advantages enumerated in the foregoing pages render it the cheapest in the end. 2d, in this respect there is a vast difference in the preparations offered for sale in this city. The one made by White, of Utica, is the one I have used the most extensively, it is darker colored, less plastic and less tenacious than the article made by J. A. Watts & Co., of the same place; it crumbles more readily, and on that account gives trouble while filling the superior teeth. Where it can be used with facility, however, it possesses all the advantages over foil that I have enumerated, though not in as great a degree as the preparation of Watts. The latter is much more plastic and tenacious. It does not waste any more than foil, and I am satisfied that it will prove in any and every case better than the best foil.

"The difference in color is sufficient to answer objection No. 3; Watts' preparation being near the shade of Abbey's foil, if any thing, a shade lighter, and less brilliant when not burnished, consequently less apt to be noticed through the enamel. As to objection No. 4, I can only state my belief, that a good operator will, after a little practice, be able to work it with more ease than he can foil, and with greater service to the patient, and as for the poor operator, it will doubtless be greatly to his patient's benefit, the very first time he uses it instead of foil; in either case, the art of using it must be acquired, no matter at what expense of time, labor and money, if its use is going to benefit our patients. And now for No. 5. The ordinary round-pointed pluggers, with grooves filed across their edges in different directions, so as to leave a number of small, well-defined points, are all that will be required for external cavities; for approximal cavities, we have only to combine the rough, condensing surface with an instrument sufficiently small as to allow of its being introduced between the teeth and into the cavity.

"There has been also before the profession a preparation of crystallized gold, made by Messrs. Taft & Watt, of Ohio. It resembles, in color and style, the article made by White; it is, however, harder, and from the specimens shown to me, I should pronounce it inferior to either of the above."

4.—*Removal of a Large Portion of Maxillary Bone.*—Dr. R. A. Miller describes in the April number of the News Letter, 1854, a case of fracture and the consequent removal of a large portion of the superior maxillary bone and floor of the antrum. The fracture was caused by the unskillful attempt of a dentist to extract the second right superior molar. "The fracture commenced immediately in front of the anterior buccal fang of the second molar, and about a third of an inch in front of the palatine fang, and extended up to, and included the floor of the antrum Highmorianum and back to the pterygoid process of the sphenoid bone." Dr. M. states that the patient is doing well.

5.—*New Plan for Removing Teeth from a Plate.*—George W. Whitaker, in the same number of News Letter, offers a new plan for the removal of teeth from plates, by pursuing which he thinks the teeth will not be cracked.

6.—*Crystallized Gold.*—In the April number of Dental News Letter, for 1854, Prof. E. Townsend gives his views on the subject of crystallized gold. He says: "The first offered to me for trial was prepared by White, of Utica, on the first of October, 1853. Prior to using it, I had its quality tested by the highest authority for standard, the assayer of the Mint of the United States, who gave me, as the result of his tests, that there was 993½-1000ths pure gold; in this respect, being equal to the foil in general use, and superior to most. My next step was to fill teeth in the hand, and after filling, to split them open. I found the gold on the surface next the dentine, to my astonishment, nearly, if not quite as hard as the surface which had received direct pressure from the instrument. I next tried filling the cavity with water, and packing the gold so as to force it out; this I did not think made so reliable a filling, and it was more difficult to give the same polish to the surface.

"To test the relative amount of gold which could be put in a given space, I filled a cylindrical cavity in a piece of ivory, so prepared, that being filled from the larger end of the tube, it could be forced out readily from the smaller; then, after filling the same as tightly as possible, and weighing the two fillings, found the crystal filling the heavier by one-fifth.

"The first experiment in the mouth was the centre of an inferior molar, a very large cavity, and the margins thin. This was filled dry, and very successfully, and in much less time than would have been required in the ordinary way.

"No. 2. *Inferior molar, buccal surface, portion of the cavity under the free margin of the gum.*—This it was impossible to keep dry, and in three weeks the patient returned, with the complaint that the filling had come out; found the lower portion had come out, removed the balance, and filled the cavity with foil. This failure I attributed to want of skill in the use of a new article.

"No. 3. *Superior bicuspid.*—Nerves removed, filled the fangs and crown; the only difficulty here was the falling from the forceps of portions into the mouth. This filling has been inspected recently, and looks well.

"No. 4. *Two superior incisors.*—Very large cavities and thin margins; both filled successfully, without cracking or injuring the front, and are as well now, four months after, as when they were finished.

"No. 5. *Very large cavity in anterior face of superior molar, passing under the free margin of the gum, and a cavity in the posterior face of the bicuspid opposite.*—After the two fillings were placed and consolidated, it was found very difficult to file between them to leave a free passage, as the gold had joined at the upper part, and become as hard as iron, or even harder.

"These are only a few of the cases in which I have used it in the last five months. I do not think sufficient time has elapsed to prove incontestibly its usefulness, or its superiority over foil in those cases where the difficulty of using foil is considered insurmountable; nor do I think it will ever supersede the use of foil as a permanent filling. Some of its best qualities for the purpose of filling weak teeth, also make it very easy to fail in making a perfect filling. Pressure upon the surface will not press it out laterally, and by so doing force it closely to the walls of the cavity. Each piece adheres to that beneath it by direct packing; and so, the very property which constitutes its safety makes it liable to be imperfect around the edges of the filling. It is necessary to pack all around the edges and make them secure, and fill the centre last. A different kind of instrument is necessary, having a rough end, sharply cut or serrated; and with a little dexterity in its use, the gold can be taken upon it and placed in the cavity without the use of forceps. There are three makers vieing for excellence in its manufacture: White, of Utica, of which I have used three ounces; in the last portion perceive a great improvement in adhesiveness, and consider it quite equal to any I have seen. Watts, also of Utica, N. Y., a chemist by profession, has been very successful in the manufacture of the article, and his gold is perhaps equal to any offered; and Taft & Watt, of Xenia, Ohio. These gentlemen are practicing dentists, and I am told intend to give the recipe to the profession when they have perfected it.

"I look upon the introduction of this article into use in the profession, as an important era, in the hope that it will soon entirely take the place of all amalgams and succedaneums for filling teeth, substituting a pure for an impure article, as I am fully persuaded there are many gentlemen in the profession who have used amalgams with the honest belief that they could do more and better for their patients' good with it than with gold foil."

7.—*Soldering full or partial Sets of Teeth.*—Dr. Hackworth offers to the profession, through the News Letter for April, 1854, his plan for soldering full or partial sets of teeth, by which, in the use of a peculiar kind of shovel, he thinks much unnecessary labor is saved. Dr. Gerhart announces, through the same number of the News Letter, two singular cases, one of a gentleman twenty-three years of age, who still had "the teeth of first dentition and none others," "the other case was that of a child two years old, whose regular lower central and lateral incisors were perfectly united."

8.—*On the comparative periods of Decay of the Bicuspid and Molar Teeth at different periods of life in the sexes.*—Dr. W. A. Pease, in the Dental Register for April, 1854, says, "that some may regard his paper as a work of supererogation, and inquire what good is to be derived from such statistics," &c.—the Dr. then, after some other general remarks goes on to give the method that he adopted in order to accumulate his facts—the plan was as follows: "When a tooth was extracted for an individual who neither required or expected to have another operation performed, it was recorded in a book kept for the purpose, wherein the age, sex, maxilla, name and part of the tooth were disease commenced, was indicated by characters. There was also regarded, with equal care, the name of the tooth, and all operations performed upon it, for all classes though in a different form; and from a cursory comparison, it is believed the period of attack will nearly coincide among the wealthy and poor, though slightly in advance of the former class. The cause of preponderance of males over females, arises from the greater attention females bestow on their teeth." After some further deductions, he give his tables, which we present to the reader, together with a part of an explanatory note by the editor of the Register.

### SUPERIOR MAXILLA—Male.

| AGE. | 1st Bic. | 2d Bic. | 1st Bic. | | 2d Bic. | | 1st Molar. | | | | 2d Molar | | | | 3d Molar. | | | | TOTALS. |
|---|---|---|---|---|---|---|---|---|---|---|---|---|---|---|---|---|---|---|---|
| | c. | c. | fs | ps | fs | ps | c | fs | ps | ls | c | fs | ps | ls | l | fs | ps | ls | |
| Under 15 | | | | | | | 6 | 3 | | 1 | | | | | | | | | |
| " 20 | | | 5 | | | 2 | 10 | 4 | 1 | 1 | 1 | | 1 | 2 | 1 | | | | |
| " 25 | | | 8 | 7 | | 20 | 22 | 19 | 10 | | 10 | 2 | 1 | 2 | 4 | 1 | 1 | 1 | 115 |
| " 30 | | | 3 | 13 | 8 | 6 | 31 | 7 | 11 | | 9 | 2 | 1 | | 11 | 7 | | 3 | 112 |
| " 35 | 1 | 1 | 8 | 2 | 8 | 4 | 13 | 3 | 1 | | 5 | 3 | | | 4 | 4 | 1 | | 58 |
| " 40 | | | 4 | 1 | 3 | 2 | 2 | 1 | 1 | | 2 | 1 | 1 | | 1 | 1 | | | 20 |
| " 50 | | | | | | | 1 | | | | | | | | 2 | | 1 | | 4 |
| | 1st bicuspid crown | 2d bicuspid crown | 1st bicuspid, front side | do. post. side | 2d bicuspid, front side | do. post side. | 1st molar crown | front side | post. side. | labial side | 2d molar crown | front side | post. side | labial side | 3d molar crown | front side | post. side | labial side | |
| | 1 | 1 | 28 | 23 | 28 | 34 | 85 | 37 | 24 | 2 | 27 | 8 | 4 | 4 | 23 | 13 | 3 | 4 | 309 |
| | | | 52 | | 63 | | 148 | | | | 43 | | | | 43 | | | | |

### INFERIOR MAXILLA.—Male.

| AGE. | 1st Bic. | 2d Bic. | 1st Bic. | | 2d Bic. | | 1st Molar. | | | | 2d Molar. | | | | 3d Molar. | | | | |
|---|---|---|---|---|---|---|---|---|---|---|---|---|---|---|---|---|---|---|---|
| | c | c | fs | ps | fs | ps | c | fs | ps | ls | c | fs | ps | ls | l | fs | ps | ls | |
| Under 15 | | | | | | 2 | 23 | 7 | 1 | | 3 | | | | | | | | 36 |
| " 20 | | | | 2 | 1 | 1 | 6 | 8 | 1 | | | 1 | | | 3 | | | | 23 |
| " 25 | | | 2 | 4 | 2 | 2 | 31 | 13 | 14 | | 21 | 6 | 2 | 2 | 8 | 4 | | 4 | 115 |
| " 30 | | | | | 3 | 7 | 23 | 13 | 8 | 4 | 17 | 4 | 2 | 1 | 19 | 3 | 2 | 6 | 112 |
| " 35 | | | | 2 | 3 | 2 | 9 | 5 | 3 | 1 | 11 | 2 | 1 | 2 | 15 | 1 | | 4 | 60 |
| " 40 | | 1 | | | 1 | 2 | 4 | 1 | | | 2 | 1 | | | 5 | 1 | 2 | 2 | 29 |
| " 50 | | | | | | | 3 | | | | | | | | | | | | 3 |
| | | 1 | 2 | 8 | 10 | 16 | 99 | 47 | 27 | 5 | 54 | 14 | 5 | 5 | 50 | 9 | 4 | 16 | 378 |
| | | | | | | | | | | | | | | | | | | | 309 |
| | | | 10 | | 27 | | 177 | | | | 88 | | | | 79 | | | | 687 |

**NOTE.—In explanation of the tables appended, which accompany Dr. Pease's article, we would say, that the letter C. stands for decay in the crown or centre of the tooth: the letter F. for front approximal, (locating the decay at that point;) the letter P. for posterior approximal; and L. for decay on the labial face of the tooth.**

### Superior Maxilla—Female.

| AGE. | Bicuspid. | | 1st Bicus. | | 2d Bicus. | | 1st Molar. | | | | 2d Molar. | | | | 3d Molar. | | | | TOTALS. |
|---|---|---|---|---|---|---|---|---|---|---|---|---|---|---|---|---|---|---|---|
| | 1st crown. | 2d crown. | f. side. | p. side. | f. side. | p. side. | crown. | f. side. | p. side. | l. side. | crown. | f. side. | p. side. | l. side. | crown. | f. side. | p. side. | l. side. | |
| Under 15 | | | | 1 | | 1 | 10 | 1 | | | | | | | | | | | 13 |
| " 20 | | | 4 | 2 | 4 | 5 | 19 | 2 | 2 | | 2 | | 1 | 1 | 1 | 1 | | | 44 |
| " 25 | | 1 | 7 | 16 | 11 | 10 | 20 | 9 | 2 | 1 | 7 | | 1 | 1 | 8 | | | 4 | 98 |
| " 30 | | | 3 | 9 | 14 | 6 | 17 | 7 | 1 | | 9 | | 1 | 1 | 12 | 1 | | 8 | 88 |
| " 35 | | | 3 | 4 | 8 | 6 | 4 | 1 | 2 | 1 | 4 | 1 | 1 | | 4 | 1 | | 3 | 43 |
| " 40 | | | 1 | | | 1 | 1 | 1 | | | 1 | | | | 3 | | | | 8 |
| " 50 | | | | | | | | | | 1 | | | | | | | | 1 | 2 |
| | | 1 | 18 | 32 | 37 | 29 | 71 | 21 | 7 | 3 | 23 | 1 | 4 | 3 | 28 | 3 | | 16 | 296 |
| | | | 50 | | 67 | | 102 | | | | 31 | | | | 47 | | | | |

### Inferior Maxilla—Female.

| AGE. | 1st Bicuspid. | | | 2d Bicuspid. | | | 1st Molar. | | | | 2d Molar. | | | | 3d Molar. | | | | |
|---|---|---|---|---|---|---|---|---|---|---|---|---|---|---|---|---|---|---|---|
| Under 15 | | | 1 | | | | 17 | 3 | | 1 | 1 | | | | | | | | 23 |
| " 20 | | | 1 | | 4 | 3 | 9 | 4 | 1 | 1 | 4 | 2 | 1 | 2 | 3 | | | | 35 |
| " 25 | | | 1 | 1 | 2 | 1 | 32 | 8 | 6 | 2 | 20 | 2 | 2 | 1 | 14 | | 1 | 6 | 99 |
| " 30 | | 3 | 1 | 1 | 3 | 7 | 24 | 9 | | 2 | 14 | 3 | 1 | 2 | 16 | 2 | | 9 | 97 |
| " 35 | | | 3 | 2 | 2 | 1 | 9 | 6 | | | 4 | 1 | | | 11 | | | 3 | 42 |
| " 40 | | 1 | 1 | 1 | | | 1 | 1 | 1 | | | | | | | | | | 6 |
| " 50 | | | 1 | | | | | | | | | | | | | | | 1 | 2 |
| | | 4 | 9 | 5 | 11 | 12 | 92 | 31 | 8 | 6 | 43 | 8 | 4 | 5 | 44 | 2 | 1 | 19 | 304 |
| | | | | | | | | | | | | | | | | | | | 296 |
| | | | 14 | | 27 | | 137 | | | | 60 | | | | 66 | | | | 600 |

9.—*Nitrate of Silver as an Application to the Teeth.*—Dr. B. T. Whitney, in the April number of the Register, says, that there has been a prejudice in regard to the use of *nitrate of silver as an application to the teeth,* which he thinks a close examination into the subject will dispel. He then describes the way the article is made, and afterwards he endeavors to prove that indilute solution will preserve animal matter. He says, "as an application to decayed or denuded teeth, that have become sensitive, I hold it in high estimation. It acts decidedly, and in a two-fold way—in destroying the animal fibres that, in their ramification through the body of the tooth, become exposed and inflamed—and then, by closing the mouth of the cells with the silver, which, in parting with its corrosive power, unites with oxygen, and forms an inert metallic oxyd. This gives a coating of insoluble metallic body over the denuded part of the tooth; though exceeding thin, yet sufficient to protect the nervous filament and dentine from irritation and contact with the 'outer world.' The tooth body being porous, absorbs more or less of the nitrate, which soon oxydizes, and gives the tooth a blackened appearance. These canals, though sufficient to transmit nutriment from the nerve pulp, through the dentine, are too minute to allow the introduction of the particles of nitrate of silver to a very great depth, so that the discoloration is superficial.

"That the oxyd of silver closes the cells, and forms a metallic surface, is perfectly demonstrable, by immersing a tooth, with the dentine exposed, in a solution of the nitrate, and then place it under the blow-pipe, with a heat sufficient to fuse the silver, when a bright silver surface will appear to the naked eye, susceptible of bearing a polish with a burnisher, almost equal to that deposited by the electro-galvanic battery upon a metal surface."

10.—*Combined Bellows, Blow-pipe and Forge.*—Dr. J. W. Baxter describes an apparatus in the Register for April, 1854, called *"combined bellows, blow-pipe and forge."* He mentions it as being "cheap and useful." This gentleman has also invented "riveting forceps" and an "artificial leech," which he describes in the same place; our space will not permit us to enter into a description at present.

11.—*Lusus Naturæ.*—Under the editorial head in the April No. of the N. Y. Dental Recorder, is mentioned the case of Miss C., about thirty-five years of age, who was possessed of two supernumerary teeth, occupying the places of the right and left superior dens sapientiæ "lusus naturæ." Dr. D. F. Lamborn reports a very singular case, a description of which we clip from the July number of the Dental Recorder.

"It is a superior third molar, with two crowns developed from three roots, and appears somewhat like the ossific union of a supernumerary with a third molar. But such is not the case, for the nerve exposed by decay of the main crown ramifies to the three roots in the same manner, which would not be the case in ossific union of two teeth. Several of my professional friends have examined the tooth, and among them Dr. B. S. Codman, of Boston, and they are of the above opinion."

12.—*Cure of Tooth-ache by Emetics.*—By Cesar Fredericq, of Ghent.—We copy the following, with the appended remarks of Dr. Wood, one of the editors, from the July number of the Southern Journal of Medical and Physical Sciences, for the present month.—*Eds.*

The pain caused by a carious tooth, observes the author, is sufficient to induce the sufferer to try every means for relief. Of all topical anti-odontalgics, creasote, as a cautery, appears to me to possess most advantage. But besides these remedies, there is one too much neglected, in my opinion: I mean, the use of emetics. Ipecacuanha, given in a vomitive dose, in case of tooth-ache, has been followed by a success wholly unexpected. It answered even in cases where the neuralgia has remained after the extraction of the tooth. Emetics constitute a valuable resource in cases of odontalgia without caries. There are many varieties of tooth-ache. It may be symptomatic of other affections, or it may be produced by an ephemeral cause. Commonly the pain is attributed to the caries; but, if so, why should not the pain be permanent in a carious tooth? Why do not people suffer continuously? Some determinate cause must be at work for the production of pain; and this varies considerably. The author believes that gastric disturbance often coincides with odontalgia, and that the close sympathy which exists between the stomach and the brain, explains why a powerful impression made on the former should exert an influence on the nerves of the head.—[*L' Observateuer Sciences Mēdicales.*]—*London Lancet.*

[In cases where creasote is useful, i. e. where the nerve is exposed to irritants, emetics can be of little avail except temporarily as a counter-irritant. In such cases, arsenious acid, "as a cautery," is always efficient, being combined with morphia and creasote. The nerve of a tooth being dead, and the pain arising from alveolar abscess or its antecedent, a cathartic, *for the constitutional remedy*, is generally preferable to an emetic. Indeed where tooth-ache is treated *constitutionally*, by remedies addressed to the *primæ viæ*, whatever its direct local cause, if aggravated by gastric disturbance, *abstinence* and *laxatives* are properly indicated, (with topical applications suited to the case,) though an emetic answering the same end, may, in certain cases, be substituted with advantage. Where the pain is properly, purely neuralgic, from general nervous irritation, (in which, of course, creasote and the like are wholly useless,) a "powerful impression" on the stomach may be effective, (at least temporarily,) by diverting the irritation, or, perhaps, benumbing the nervous sensibility of this organ, supposing it to be the seat and source of the irritation.]

### ANATOMY AND PHYSIOLOGY.

13.—*Influence of Respiration on the Pulse.*—Dr. S. W. Mitchell publishes an interesting paper on this subject in the *American Journal of Medical Sciences for April.*

His results are not confined to the ordinary subjects of study in this connection, but embrace the various changes consequent upon the respiratory movements.

As a preliminary step, he investigated the influence of mental attention on the heart's action. He found that the pulse was slightly accelerated, but not sufficiently so to interfere with his results. Study or close application of the mind renders the pulse less frequent.

Inspiration diminishes the frequency of the pulse, and sometimes a very forcible inspiration seems actually to suspend the heart's action. Expiration, on the other hand, quickens the circulation, and these effects are most marked as the respiratory movements approach their extreme limits.

In a careful analysis of forty cases, it appears that the diminution of pulsation during inspiration was 10.5 beats in 80.5, or 13.17 per cent., while its increase during expiration was 12.6, in the same number, or 15.65 per cent. on the normal standard. The average difference between the extremes of respiration was 23 beats. In one case there was no fall of pulsation during inspiration, but in all there was a rise during expiration. The greatest increase observed in expiration, was 35 over 81, the natural standard; the smallest was 1 over 89. The greatest relative decrease observed, in inspiration, was 24 in 82. The greatest absolute decrease was 28 beats, the normal pulse being 96. It is to be observed, that the mobility is not the same for expiration and inspiration. Thus, the patient who only gained 1 beat in expiration, lost 16 in inspiration, while the one who lost nothing in inspiration gained 12 in expiration. The smallest difference between the extremes was 11, the greatest 44 beats.

The respiratory capacity of the lungs does not appear to exert any influence over the change of rapidity.

As an illustration of the physical cause of these changes, the writer suggests the figure of an elastic bag, with elastic tubes coiled about its walls. The bag being distended, these tubes are stretched and diminished in calibre. In the lungs, the vessels are distributed over the walls of the cells. When these last are distended, as in inspiration, the capillaries are stretched, and perhaps compressed.

To determine this point experimentally, carbonate of soda was injected into the pulmonary vessels of a living animal, after which, all the thoracic viscera were removed and placed in a solution of the same salt. A long glass tube was then fitted into the pulmonary artery, another into the left auricle, and a third, provided with a stop-cock, into the opening of the trachea. Dilute serum was then poured into the tube of the artery, till a bloody liquid rose from that inserted into the vein. The lung now being inflated, the level of the fluid fell in both tubes, most in that inserted into the artery; when the inspiration was forced and permanent, the circulation ceased entirely. During expiration, the column in the pulmonary artery fell, till both columns stood at the same level.

In vivisections, it was observed that the lung always flushed during expiration, and became pale on inspiration. When the lung was slightly wounded during full inspiration, no hemorrhage occurred, but the lung bled during expiration. The bleeding was always more copious during expiration, provided no large vessel had been wounded.

The author draws the following inferences:

1. "It seems to me clear enough, that the pulmonary circulation is modified by the various conditions of respiration, in which the lungs may be placed. Do not, relatively, similar effects attend every respiratory movement, however simple? We cannot demonstrate this upon the healthy living lung, yet the inference would seem to be a fair one.

"2. During complete inspiration, the tide of blood is momentarily retarded in the capillaries of the lung. Aeration of the vital fluid then takes place with the greatest facility; and during expiration, and more especially in complete expiration, the blood thus fully aerated is expelled from the lungs by the rapidly acting heart. In other words, the circulation in the lungs is slower when these organs contain most air, and becomes most easy and rapid during the movement of expiration.

"The effect of respiration in mechanically diverting the blood from the course of the fœtal circulation, is also of interest in this connection."

14.—*Cellulose in the Human Body*—A very remarkable discovery was made by Purkinje, when he saw bodies in the human brain which resembled granules of starch, and corresponded to that vegetable substance in their relations to chemical reagents.

Virchow has lately examined them, and has ascertained that they possess all the chemical properties of cellulose, a substance hitherto regarded as characteristic of vegetable organisms. Iodine colors them light blue, and hydrated sulphuric acid gives the violet tint which forms the specific characteristic of vegetable cellulose.

They belong, he says, exclusively to the ependyma, and are found only in the superficial layers of the ventricles, in the spinal marrow, particularly the central gray substances corresponding to the ependyma of the obliterated canal, and in the nerves of the senses, as in the gray substance of the olfactory nerve other concentric corpuscles from organs included without the cranium and spinal column, show no such reaction.

These corpuscles have since been sought for in various parts of the body without success. In waxy spleen, however, Virchow has found similar bodies, the result of a transformation of the Malpighian glands into grains resembling boiled sago. Iodine and sulphuric acid especially produce the characteristic reaction of cellulose.

15.—*On the Movements of the Glottis in Respiration.*—Dr. John C. Dalton, Jr., contributes to the American Journal of Medical Science, an article with the above caption.

He has proved satisfactorily by experiments on living animals that "there is, during normal respiration, a constant and regular motion of the vocal cords, by which the size of the glottis is alternately enlarged and diminished, synchronous with the inspiratory and expiratory motions of the chest. These movements are of the same character as the general automatic motions of respiration, of which they in fact form a part. It is at the same time, and by the same nervous influence, that the chest expands to inhale the air, that the glottis opens to admit it. When the chest collapses, the glottis returns to its original size, and the air is expelled through it from below."

The separation of the chords and the consequent opening of the glottis is effected by the action of the posterior circo-arytenoid muscles, which rotate the arytenoid cartilages outward and backward and at the same time draw them backward, so that the opening of the glottis is at the same time lengthened and widened. The motion can be produced in the recently killed calf by irritating the first terminal branch of the inferior laryngeal nerves which furnish the motor power. A section of these nerves instantly puts a stop to the motions under consideration, and the chords of the glottis are closed like a valve by the column of air.

These nerves being given off by the pneumogastric within the chest, have their function necessarily impaired when the main trunk is cut in the neck, so that paralysis of the glottis is one of the consequences of this operation. To determine the difference between a section of the main trunk and its recurrent branch, two dogs were selected, in one of which, (No. 1,) the pneumogastrics was cut, and in the other, (No. 2,) the inferior laryngeals. The only marked difference in the symptoms was the diminution in the frequency of the respiratory movements in the animal that had its *par vagum* cut. In the other, the respirations continued frequent and laborious to the end. Both died between 30 and 40 hours after the operation. Both had the same congestion of the lungs. No. 1 had his blood coagulated and the abdominal organs natural, while No. 2 had the blood fluid and the abdominal organ congested.

"This experiment," says the observer, "affords additional evidence of the two following facts, which have been already more or less generally acknowledged by experiments:

"*First*. After section of the pneumogastrics, death is produced by congestion of the lungs.

"*Second*. This congestion is not a direct effect of the division of the nerves, but is caused by the imperfect admission of air into the chest."

16.—*Elasticity of Arteries, a Source of Animal Heat.*—The Medical Times and Gazette for May 27th, contains a paper from Dr. Winn, calling attention to the influence on the production of animal heat of the elastic recoil of the arteries.

He shows that there is a *residual heat,* not accounted for by physiologists on the basis of the oxydation of carbon and hydrogen in the system. His attention was called to the possibility of the arteries producing heat, by some experiments which he had made upon caoutchouc. To determine the fact, he had recourse to direct experiment.

He took about an inch of a bullock's aorta, laid it open, stripped it of its inner and outer tunics, and by alternately stretching it and allowing it to contract, he succeeded in developing sufficient heat to raise the thermometer two degrees in as many minutes. He took the necessary precautions against the influence of external heat. He muffled his hands in a double wrapper of flannel, covered his mouth with a handkerchief, to prevent the warm breath from influencing the thermometer, and performed the experiment in a room without a fire, at 55°. To prevent evaporation, he dried the artery as much as possible, with a cloth.

17.—"*The Process of Repair in Teeth.*—The recent number of *Guy's Hospital Reports,* contains a very interesting communication by Dr. S. J. A. Salter, on the laws which regulate the formation of the "Dentine of Repair," one of the forms of what has been called secondary dentition, or that after formation by which the pulp cavity of the tooth is diminished or obliterated, after the tooth has attained a mature and adult condition.

"There are three forms of secondary dentine: *Osteodentine,* in which the new tissue is arranged in systems resembling the Haversian systems of bones around isolated blood-vessels; the dentinal tubes radiating from each centre. It always occurs in states of irritation or inflammation of the pulp. *Dentine excrescences* are little nodules of secondary dentine, occasionally found attached to the interior of the pulp cavity of otherwise healthy teeth. *Dentine of repair* is the special subject of the paper. This deposit is thrown out within the pulp cavity, *opposite* to that part of the external surface of the tooth where a fracture or wearing of the original dentine has taken place, thus thickening the body of the tooth opposite the injured part, so that teeth which are worn down even level with the gum still present no cavity.

"This process corresponds with the most beautiful exactness to the external lesion; as long as the enamel only is injured, no dentine of repair is deposited; but as soon as any of the dentine tubes are broken off or worn away on the surface of the tooth, so soon is there thrown out at their opposite extremities towards the pulp a deposit, limited with the utmost exactness to the injured tubes; not mathematically opposite, therefore, to the injured part, but *physiologically* opposite, according to the wavy course of the tubes. The dentine of repair is clear and translucent, and the part of the original dentine involved in the process becomes also more transparent than usual, in consequence of its tubules being filled up with solid matter."

18.—*Relations of Fat and Sugar.*—Dr. Gibb read a paper before the physiological section of the Medical Society of London, on the subject of the relation of the fat in the liver to its sugar.

He found that, in birds, those which had the most fat, had also the most sugar in the liver. Among the mammalia, those which possessed but little fat in this organ had also little sugar there, while those, which, like seals, had the liver gorged with fat, also contained large quantities of sugar in the same organ.

19.—*Phosphate of Lime in Nutrition.*—The Virginia Medical and Surgical Journal contains an abstract of a paper by Bouchardat, on some recent discoveries of Mouriés, a young French chemist, in the part played in nutrition by phosphate of lime.

His conclusions are the following:

"1. Phosphate of lime plays a more important part in nutrition than has heretofore been believed. Independently of its necessity as a constituent of bone, this salt maintains that irritability without which there is no assimilation, and consequently no nutrition. Its insufficiency, therefore, produces death, with all the symptoms of inanition, while its insufficiency in a less degree produces a series of lymphatic diseases.

"2. The food consumed in cities is deficient in this respect. Nurses' milk has, consequently, the same defect. The infant, as well as the fœtus, suffers from the deprivation of this element, so indispensable to its development and life. Hence one of the causes of the increase of the number of still-born children, and of the mortality of infancy.

"3. The addition of this salt, in combination with animal matter, to alimentary substances, obviates one cause of disease and death."

The report goes on to show that different species of animals require different proportions of this salt, but that the proportion is constant for each species. It also reveals the remarkable fact of a close relation subsisting between the amount of phosphate of lime in the blood and the normal temperature of the animal, from which the author infers that this salt keeps up animal irritability, without which nutrition is impossible. One of the tables is so curious that we cannot refrain from copying it. The temperature is rated by the centigrade thermometer, now commonly used in scientific observations.

| | Phosphate of Lime. | Temperature. |
|---|---|---|
| Blood of the duck, | 1.50 | 42.5° |
| " " hen, | 1.35 | 41.5 |
| " " pigeon, | 1.20 | 40. |
| " " man, | .80 | 37.5 |
| " " horse, | .40 | 36.8 |
| " " frog, | a trace | 9. |

If these tables are correct, it is impossible to avoid the conclusion that this salt plays an important part in the generation of animal heat.

The amount of phosphate of lime necessary to repair the waste of the system is then estimated, upon the basis of the excretions, as 110 grains daily. Women in the country discharge, according to this chemist, 90 grains of this salt in their urine, whereas the quantity in the secretion of the kidneys in city women varies from 20 to 90 grains in the 24 hours. In 18 healthy country women, the milk contained from 1.2 to 2.4 per cent. of earthy phosphates, while, in 10 Paris nurses, the proportion varied from 0.5 to 0.9 per cent., and in 7 others there was but a trace of this salt.

The results of a mode of treatment consisting in supplying phosphate of lime artificially were favorable. In 13 cases, having an average of 0.7 per cent. of phosphate of lime, 75 grains with twice the quantity of albumen was daily administered in soup; in a week the proportion of earthy phosphate rose to 2.1. Five pregnant women were subjected to the same treatment; after delivery their milk contained from 1.9 to 2.1 per cent. of phosphate of lime. Only 3 of the 18 children died.

If these statements are true, as they probably are, since they are brought forward under the auspices of Bouchardat, our readers will be able to see the connection between them and the very important fact of the rapid decay of the teeth in some sections of this country.

### EDITORIAL DEPARTMENT.

